# The Development of Functional Overreaching Is Associated with a Faster Heart Rate Recovery in Endurance Athletes

**DOI:** 10.1371/journal.pone.0139754

**Published:** 2015-10-21

**Authors:** Anaël Aubry, Christophe Hausswirth, Julien Louis, Aaron J. Coutts, Martin Buchheit, Yann Le Meur

**Affiliations:** 1 Laboratory of Sport, Expertise and Performance (INSEP), EA, 7370, National Institute of Sport, Expertise and Performance, Paris, France; 2 Research Institute for Sport and Exercise Sciences, Liverpool John Moores University, Tom Reilly building, L3 3AF, Liverpool, United Kingdom; 3 Sport and Exercise Discipline Group, Faculty of Health, University of Technology Sydney (UTS), Sydney, Australia; 4 Institute of Sport, Exercise and Active Living (ISEAL), School of Sport and Exercise Science, Victoria University, Melbourne, VIC, Australia; University of Rome Foro Italico, ITALY

## Abstract

**Purpose:**

The aim of the study was to investigate whether heart rate recovery (HRR) may represent an effective marker of functional overreaching (f-OR) in endurance athletes.

**Methods and Results:**

Thirty-one experienced male triathletes were tested (10 control and 21 overload subjects) before (Pre), and immediately after an overload training period (Mid) and after a 2-week taper (Post). Physiological responses were assessed during an incremental cycling protocol to exhaustion, including heart rate, catecholamine release and blood lactate concentration. Ten participants from the overload group developed signs of f-OR at Mid (i.e. -2.1 ± 0.8% change in performance associated with concomitant high perceived fatigue). Additionally, only the f-OR group demonstrated a 99% chance of increase in HRR during the overload period (+8 ± 5 bpm, large effect size). Concomitantly, this group also revealed a >80% chance of decreasing blood lactate (-11 ± 14%, large), plasma norepinephrine (-12 ± 37%, small) and plasma epinephrine peak concentrations (-51 ± 22%, moderate). These blood measures returned to baseline levels at Post. HRR change was negatively correlated to changes in performance, peak HR and peak blood metabolites concentrations.

**Conclusion:**

These findings suggest that i) a faster HRR is not systematically associated with improved physical performance, ii) changes in HRR should be interpreted in the context of the specific training phase, the athletes perceived level of fatigue and the performance response; and, iii) the faster HRR associated with f-OR may be induced by a decreased central command and by a lower chemoreflex activity.

## Introduction

Increases in training intensity and volume are typically undertaken by athletes in an attempt to enhance physical performance. However, when the balance between appropriate training stress and adequate recovery is disrupted, an abnormal training response may occur and *functional overreaching* (f-OR) may develop. Intensified training can then result in a decline in performance; however, when appropriate periods of recovery are provided, a ‘‘supercompensation” effect may occur with the athlete exhibiting an enhanced performance compared with baseline levels. When this ‘‘intensified training” continues, the athletes can evolve into a state of extreme OR or non-functional overreaching (nf-OR) which will lead to a stagnation or decrease in performance that will not resume for several weeks or months. In contrary to the nf-OR stage, f-OR is often intentionally induced by coaches–typically through periods of intensified training such as training camp periods–with the intent that the temporary performance decrement is followed by a taper-induced performance supercompensation [1]. However, despite its popularity, the evidence supporting a maximized training response after deliberate f-OR is not clear [1]. Indeed, Aubry et al. [2] recently showed that triathletes who became f-OR following a 3-week overload training program had a poorer taper-induced performance response compared to a group of well-trained triathletes who completed the same intensified training without the signs and symptoms of f-OR. Additionally, the competitive schedules in many sports (*e*.*g*., triathlon, cycling, and cross-country skiing) can require athletes to compete in a series of events over several consecutive weeks or months, making it difficult for athletes to maintain optimal performance throughout the season. During these periods, it can be challenging to deliver training programs that meet the need to recover from competition to prevent f-OR whilst also allowing athletes to maintain optimal fitness levels.

The currently accepted method for detecting f-OR is to assess changes in physical performance following a resting period of several days or weeks, and the diagnosis is confirmed when performance is decreased and the athlete perceived high fatigue [1]. However, this method is not well accepted by coaches and athletes because it may disrupt the planned training schedule and lead to detraining. It is therefore important to identify markers of f-OR that can effectively identify athletes who are not adapting to training, but also be easily applied without interfering with the training process. Among the different biological markers [3], heart rate (HR) has been suggested as a promising tool for monitoring if athletes are adapting to training [4]. For example, a reduced HR response has been observed during standardized exercise bouts in f-OR endurance athletes. Moreover, Le Meur et al. [5] used a multifactorial analysis including physiological, cognitive, biomechanical, and perceptual parameters to show that the decrease in HR at submaximal intensities and at exhaustion was the most discriminating response between f-OR athletes and control athletes. Additionally, others have reported a decreased maximum HR in f-OR endurance athletes [6–8]. Le Meur et al. [9] recently extended this work to show that ‘chronotropic incompetence’ with f-OR was associated with a reduced cardiac output and a reduced adrenergic response at both maximal and submaximal exercise.

Another cardiac parameter often used to infer the ability to physically perform is heart rate recovery (HRR) after a standard exercise bout [4, 10]. HRR is defined as the rate at which HR declines, usually within the first minute after the cessation of physical exercise. The HRR is influenced by exercise intensity, the direct autonomic nervous action upon the heart (i.e. withdrawal of the sympathetic nervous system and up-regulation of the parasympathetic reactivation) and the clearance of stress metabolites (i.e. plasma catecholamines, lactate, H+, Pi, etc.) [11]. Generally, a faster HRR is considered as to indicate a faster post-exercise recovery and a better aerobic fitness [10]. A few studies have investigated the effects of heavy physical training load on HRR response in endurance athletes suspected of f-OR [12–15]. Unfortunately, these studies have provided conflicting results, with some reporting decreased HRR [14] whilst others showing faster HRR [12, 13, 15] in athletes suspected of f-OR. Additionally, these studies have not reported a decrease in individual performance in all participants in response to overload training, making it difficult to delineate the effects of overload training effect from true f-OR.

The aim of the present study was to clarify if the use of HRR could facilitate the diagnosis of functional overreaching in endurance trained athletes, while controlling the above mentioned methodological issues. More precisely, the present research was designed to: 1) examine the specific HRR responses to overload between a control group and two groups developing acute fatigue (i.e. no performance decrease despite high perceived fatigue, AF) compared to f-OR during an overload training period; and, 2) examine some of the possible determinants of changes in HRR. Therefore, we re-visited known data sets that have characterized both cardiac response and catecholamines excretion at exercise in such kind of population [9]. We extended this analysis to the post-exercise recovery period to investigate the HRR response and its potential modulating physiological factors.

## Materials and Methods

### Subjects

Forty well-trained male triathletes volunteered to participate in this study. Their performance level over the Olympic distance triathlon (*i*.*e*., 1.5-km swimming; 40-km cycling; 10-km running) ranged between 2 h and 2 h 20 min (mean performance: 131 ± 5 min, regional to national level of competition). The experimental design of the study was approved by the Ethical Committee of Saint-Germain-En-Laye (acceptance no. 12048) and was conducted in accordance with the Declaration of Helsinki. Before participation, subjects underwent medical assessment by a cardiologist to ensure normal electrocardiograph patterns and obtain a general medical clearance. All subjects were free from chronic diseases and were not taking medication at the commencement of the study. After comprehensive verbal and written explanations of the study, all subjects gave their written informed consent to participate.

The triathletes were randomly assigned to either the control group (n = 10) or the overload training group (n = 22) according to a matched group experimental design on the basis of performance level, habitual training volume, and past experience in endurance sports. All subjects had regularly competed in triathlons for at least 3 y and were training a minimum of 10 h^.^wk^-1^.

### Study design

**Fig 1:**
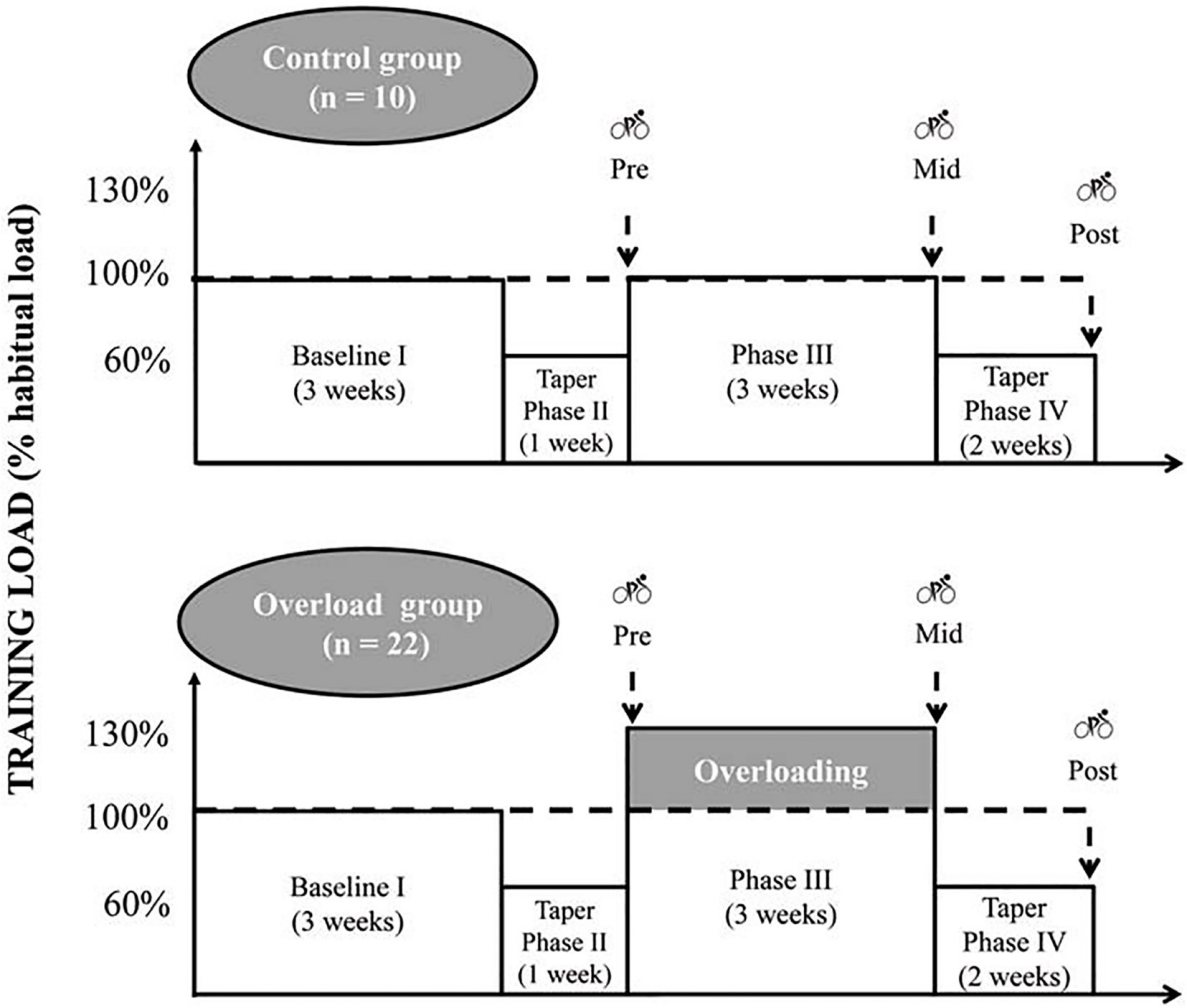
Schematic representation of the experimental protocol. Bicycle symbols represent maximal incremental cycling test.

An overview of the study design is shown in Fig 1. The training of each triathlete was monitored for a period of 9 wk, which was divided into four distinct phases. The two first phases were similar for all subjects. During the first phase (I, 3 weeks), the triathletes completed their usual training. The second phase (II) consisted of 1-wk of moderate training load during which the subjects were asked to reduce their habitual training volume by approximately 40% while maintaining the training intensity. These tapering strategies were selected according to the guidelines for optimal tapering in endurance sports [16]. During the third period (III), the overload training group completed a 3-wk overload program designed to deliberately overreach the subjects: the duration of each training session of the classic training period was increased by 30% (*e*.*g*. a training including 7 repetitions of 1000 m completed in 3-min 30-s during a 1-h session was converted into an 80-min session including 9 repetitions of 1000 m completed in 3-min and 30-s). The subjects reproduced the same training program during each week of the overload period, so that both the content and the weekly distribution of the training session remained consistent. The control group repeated its usual training program during this period. Thereafter, all the participants completed a two-week taper period (fourth experimental period, Taper), where their normal training volume (first phase) was decreased by 40% each week (*e*.*g*. the same training including 7 repetitions of 1000 m completed in 3-min and 30-s during a 1-h session was converted into a ≈ 35-min run including 4 repetitions of 1000 m completed in 3-min and 30-s).

During phase I, the subjects reported to the laboratory to become familiarized with the testing protocols used during the study (described below). A maximal incremental cycling test was performed on three occasions (Fig 1), including Pre (*i*.*e*., after phase II), Mid (*i*.*e*., after phase III), and Post (*i*.*e*., after phase IV), respectively. For each subject, the test was completed on the same day of the week at the same time. To ensure that any performance variations during the maximal incremental tests were due to the global training regimen and not the training session(s) performed on the previous day, the subjects were required to abstain from training during a 24-h period before each testing session. During the 48 h before each test, the triathletes were also required to follow nutritional and hydration guidelines designed to ensure adequate muscle glycogen store resynthesis and a well-hydrated state prior to each test. Specifically, all participants were instructed to eat until satiety was reached at each meal. Breakfast consisted of a variety of macronutrients from both solid and liquid energy sources. The selected foods included an assortment of cereals, bread, fruit, yogurt, milk, juice, ham, and cheese. For lunch and dinner, the subjects consumed a mixed salad as starter, then white meat during lunch and fish during dinner. The side plate consisted of a mixed of 50% carbohydrates (*i*.*e*., pasta, nice, and noodles) and 50% of vegetables (i.e., green beans, broccoli, and tomatoes). One piece of fruit and tub of yogurt were added as dessert, at both lunch and dinner. The subjects were also asked to drink more if they observed their urine to be dark and were reminded of these recommendations before each test by e-mail or phone call. No data was collected to assess if participants complied with the instructed dietary intake across all visits. The compliance was only controlled by verbal feedback.

### Measurements

#### Perceived fatigue

Before exercise testing, subjects were asked to complete the profile of mood state (POMS) questionnaire to assess perceived fatigue [17]. This questionnaire was chosen because it has been found to be sensitive for detecting f-OR [18].

#### Peak power output and maximal oxygen uptake

Maximum oxygen uptake ($\dot{\text{V}}$O_2max_) was assessed on an electronically braked cycle ergometer (Excalibur Sport, Lode®, Groningen, The Netherlands) equipped with standard 170-mm cranks where the athletes used their own shoes. The seat and handlebar positions were adjusted to the specific requirements of each athlete and were standardized between sessions. The maximal test was performed until complete exhaustion which was confirmed by the criteria described by Howley et al. [19]—that is, a plateau in $\dot{\text{V}}$O_2max_ despite an increase in power output (i.e. no change or a drop in $\dot{\text{V}}$O_2_ for an increase in power output), a respiratory exchange ratio value of 1.15, or a HR over 90% of the predicted maximal HR. This exercise protocol started with a warm-up of 5 min at a workload of 100 Watts (W), followed by 5 min at 150 W and 5 min at 200 W. Thereafter, further increments of 25 W were added every 2-min until volitional exhaustion. Subjects wore a face mask covering their mouth and nose for breath collection (Hans Rudolph, Kansas City, MO, USA), and the expired oxygen and carbon dioxide concentrations were continuously measured breath-by-breath (Quark, Cosmed, Rome, Italy). The gas analysers and the flowmeter of the applied spirometer were calibrated prior to each test.

After the test, breath-by-breath values were visually inspected and averaged over 30-s. The highest 30-s average value was taken as $\dot{\text{V}}$O_2max_. Peak power output (PPO) was calculated as Wcompl + 25 × (t/120); Wcompl is the last completed workload, and t is the number of seconds in Wcompl.

#### Blood lactate concentration

A fingertip blood sample (5 *μ*L) was collected and blood lactate concentration ([La^-^]) was determined (Lactate Pro, Arkray, Kyoto, Japan) at the end of each cycling stage, immediately at exercise cessation and each 90 s until [La^-^] reached its peak value. The accuracy of the analyzer was checked before each test using standards. The suitability and reproducibility of this analyzer have been previously established throughout the physiological range of 1.0–18.0 mmol^.^L^-1^ [20].

#### Heart rate

An impedance cardiography device (Physioflow type PF05L1, Manatec Biomedical, Macheren, France) [21] was used in this study to determine peak heart rate (HR_peak_) at the end of maximal incremental test and HRR in the first minute of post exercise recovery directly after the cessation of exercise. The Physioflow was carefully calibrated before each test according to the manufacturer recommendations. HRR was assessed during the recovery period after cessation of the performance test and reported as both the absolute (in beat per minute) and the relative (% of HR_peak_) difference between the HR at cessation of exercise and the HR recorded at 60-s during recovery in sitting position on the ergocycle. This HRR parameter was selected because it is easy to collect in the field and its reliability has been demonstrated to be higher than other HRR measures [22].

#### Blood catecholamines

A 22-G catheter was inserted into an antecubital vein while the subjects rested quietly in a chair ≈30-min before the maximal cycling test. Subjects then rested for 10-min after catheterization to allow for normalization of catecholamine levels resultant from the catheter insertion. Approximately 6 ml of blood were obtained and placed into ethylenediaminetetraacetic acid tubes immediately after exercise cessation. The tubes were immediately placed on ice and centrifuged within 5 min of collection. The plasma and the serum samples were aliquoted and stored at -80°C until analysis. Plasma norepinephrine ([NEp]) and epinephrine ([Ep]) concentrations were determined using high-performance liquid chromatography with electrochemical detection. All samples were analyzed the same day for each participant.

#### Perceived exertion

The rating of perceived exertion (RPE) was measured verbally using the Borg 6–20 scale [23] immediately at the end of the maximal cycling test. The subjects were anchored with the scale prior to testing and reminded of the appropriate use prior to each incremental test throughout the experiment.

### Training monitoring

Training volume and intensity were calculated on the basis of recordings from HR monitors (Polar, Kempele, Finland). For all subjects, HR was measured every 5-s during each training session throughout the entire protocol. The training intensity distribution was calculated using three HR zones: *1*) ≤HR at 2 mmol^.^L^-1^; *2*) between HR at 2 mmol^.^L^-1^ and HR at LT; and *3*) HR values above HR at LT. Given that the relationship between [La^-^] and HR values during exercise can be influenced by a heavy training load program [5], the reference HR values were reassessed after each incremental cycling test.

### Data Analysis

#### Assessment of overreaching

The subjects in the overload training group were distributed a posteriori into two subgroups according to their response to the overload period and during the subsequent taper. substantially decreased PPO and increased perceived fatigue at Mid with subsequent performance supercompensation at Post were allocated to the f-OR group [1]. To be diagnosed as f-OR at Mid, athletes of the overload training group had to report an increased perceived fatigue and to show a individual performance decrement larger than 0.3 of the typical performance variation (i.e. threshold for small effect size, 16) during the maximal cycling test, assessed in the control subjects (changes in Pre to Mid performance values, without training load manipulation). The remaining subjects in the overload training group, who maintained or increased their performance after phase III despite high perceived fatigue, were considered to experience only AF [1].

#### Statistical Analysis

Data were analyzed using a magnitude-based inference approach for all parameters [24]. To account for the effect of peak HR on HRR response, the between-group differences in HRR change were also conducted with the change in peak HR used as covariate. All data were log transformed before analysis to reduce bias arising from non-uniformity of error. For clarity, however, the values presented in the text and figures are not transformed. The magnitude of the within-group changes, or between-group differences in the changes, were interpreted by using values of 0.3, 0.9, 1.6, 2.5 and 4.0 of the within-athlete variation as thresholds for small, moderate, large, very large, and extremely large differences in the change between the trials [24]. Quantitative chances of higher or lower values than the smallest worthwhile change were evaluated qualitatively as follows: <1%, almost certainly not; 1%–5%, very unlikely; 5%–25%, unlikely; 25%–75%, possible; 75%–95%, likely; 95%–99%, very likely; and >99%, almost certain. If the chance of higher or lower values was 95%, the true difference was assessed as unclear. Otherwise, we interpreted that change as the observed chance [24]. The practical interpretation of an effect was deemed unclear when the 90% confidence interval (CI) of standardized change/difference included zero [24, 25]. Pearson’s product-moment correlation analysis was also used to compare the association between individual changes in HRR (ΔHRR) during the overload period and changes in blood metabolites concentrations (i.e. Δ[La^-^], Δ[Ep], Δ[NEp]). The following criteria were adopted to interpret the magnitude of the correlation (r) between test measures: ≤ 0.1 trivial, < 0.1–0.3 small, < 0.3–0.5 moderate, < 0.5–0.7 large, < 0.7–0.9 very large, and < 0.9–1.0 almost perfect. If the 90% confidence intervals overlapped small positive and negative values the magnitude was deemed unclear; otherwise that magnitude was deemed to be the observed magnitude [24].

## Results

During the 9-wk experimental period, eight subjects (two and six for control and the overload groups, respectively) did not follow the prescribed training due to injury or personal reasons and were excluded from subsequent analyses. The final samples were *n* = 10 and *n* = 22 for control and the overload groups respectively.

### Assessment of the OR

**Fig 2:**
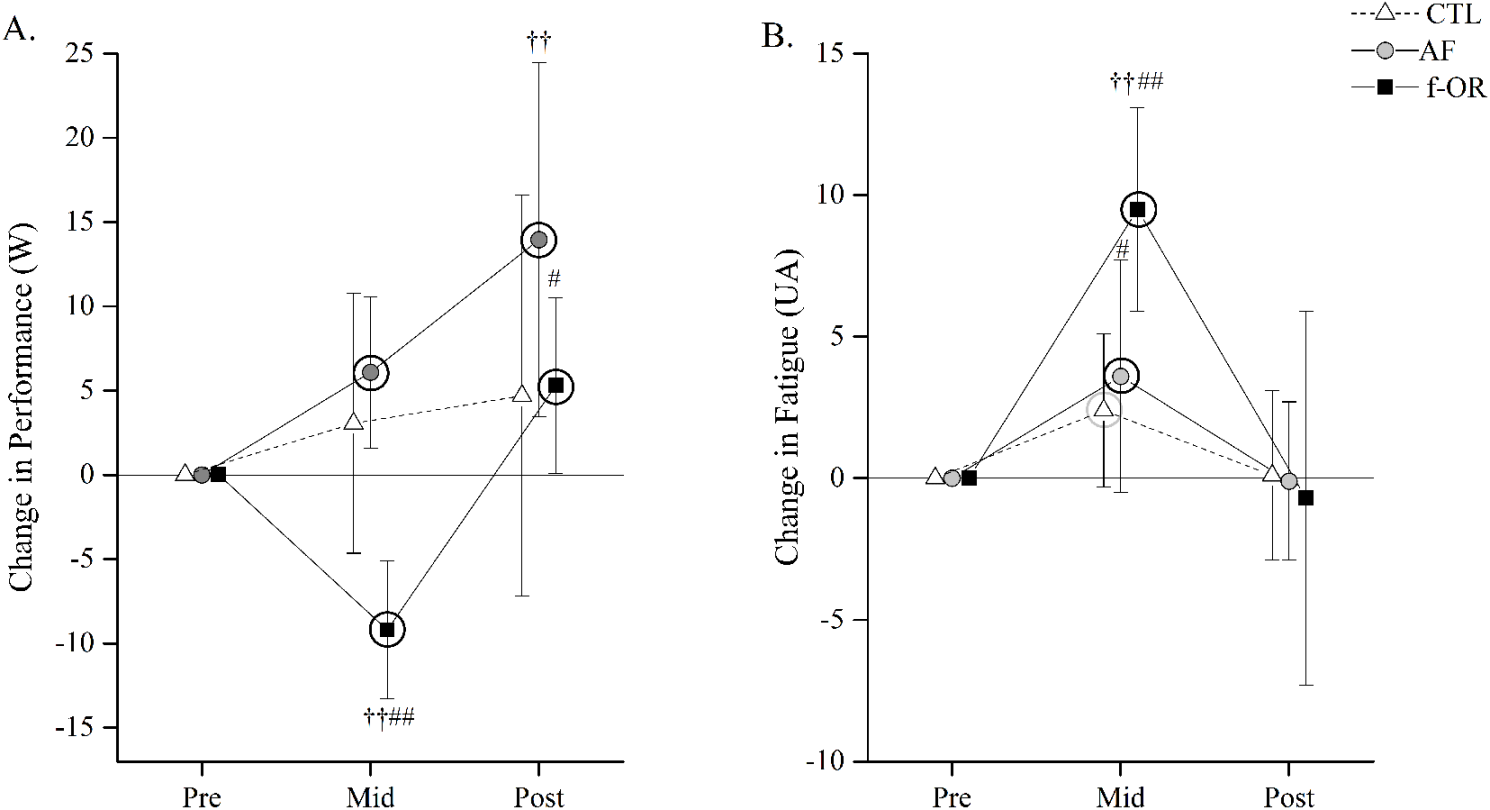
Changes in peak power output (A) and perceived fatigue (B) during the maximal incremental cycling test (mean ± 90% CI). Grey and black circles around symbols denote likely (*i*.*e*., 75%–95% chances that the true value of the statistic is practically meaningful) and very likely to almost certain (*i*.*e*., > 95% chances that the true value of the statistic is practically meaningful) within-condition difference from baseline (Pre), respectively. Between-group difference in change from Pre vs. control, #likely; ## very likely to almost certain. Between-group difference in change from Pre vs. AF, †likely; †† very likely to almost certain. AF: acute fatigue; f-OR: functional overreaching.

At baseline, all subjects reported low fatigue level at rest (*i*.*e*., all subjects reported “not at all” or “a little” on the POMS fatigue item at Pre), assuming that they were not in an OR state. Ten of the 22 overload subjects demonstrated a substantial decrease in performance after the overload period (372 ± 36 vs 363 ± 36 W at Pre and Mid, respectively, almost certain moderate decrease, Fig 2A) followed by a very likely small performance supercompensation during the taper (378 ± 36 W at Post, Fig 2A, S1 Fig). For these ten subjects (Table 1), the performance decrement reached the “OR threshold” at Mid (-0.6% of performance value at Pre). The f-OR subjects reported a concomitant almost certain very large increase in perceived fatigue (4 ± 4 vs 13 ± 5 W at Pre and Mid, respectively, Fig 2B, S1 Fig). One subject from the overload group demonstrated a decrease of performance associated with high perceived fatigue at Mid, but his performance only restored during the taper (i.e., without supercompensation). This triathlete was diagnosed as ‘‘non f-OR” [1] and was excluded from subsequent analyses. The 11 other subjects in the overload group, who preserved their performance level after the overload period despite a very likely moderate increase in perceived fatigue (4 ± 3 at Pre vs 9 ± 5 at Mid, Fig 2B), were diagnosed as AF. Thus, the subsequent results are presented for 10 f-OR subjects (f-OR group), 11 AF subjects (AF group), and 10 control subjects (Table 1). Between-group differences in performance at baseline were unclear.

**Table 1:** Age, competitive experience (experience), maximal oxygen uptake ($\dot{\text{V}}$O_2max_) and maximal aerobic power (MAP) before (Pre) the intervention period for the control, the acute fatigue (AF) and the functionally overreached groups (f-OR).

	Control (*n* = 10)	AF (*n* = 11)	f-OR (*n* = 10)
**Age (years)**	37 ± 6	33 ± 6	36 ± 5
**Body mass (kg)**	75 ± 7	73 ± 7	73 ± 9
**Body height (cm)**	183 ± 6	178 ± 6	180 ± 6
**Experience in endurance sport (years)**	13 ± 11	15 ± 7	12 ± 6
**MAP (W)**	355 ± 26	357 ± 28	372 ± 36
$\dot{V}$ **O**_**2max**_ **(mLO**_**2**_ **·min** ^**-1**^ **)**	4300 ± 359	4359 ± 486	4543 ± 416

Values are mean ± SD. Between-group difference at baseline were *unclear* for all parameters.

### Compliance to the Training Program

Changes in weekly training volume, the distribution of the relative training time spent in the intensity zones, and the number of training sessions per training phase in the three disciplines during the four phases of the protocol are presented in Table 2.

**Table 2:** Weekly average training volume (mean ± SD), distribution of training time in the intensity zones (see Methods) and number of training sessions per week in swimming, cycling and running during the 9 weeks protocol.

Parameters	Groups	Baseline(I)	Taper(II)	Overload(III)	Taper(IV)
Duration (weeks)		3	1	3	2
**Weekly volume (hours)**	Control	12 ± 3	6 ± 1**	12 ± 2	6 ± 1**
	Acute Fatigue	13 ± 3	7 ± 1**	17 ± 3** ^##^	7 ± 1**
	f-OR	14 ± 3	7 ± 1**	19 ± 3** ^##^	7 ± 1**
**Distribution of training intensity in zones 1/2/3 (% of total training time)**	Control	62/30/8	67/26/7	66/26/8	63/28/9
	Acute Fatigue	65/26/9	65/26/9	65/27/8	61/29/10
	f-OR	64/30/7	68/26/6	68/26/6	64/28/8
**Weekly number of swimming, cycling, running sessions**	Control	3/3/3	2/3/3	3/3/3	2/3/3
	Acute Fatigue	3/3/3	2/3/3	3/3/3	2/3/3
	f-OR	3/5/3	2/4/3	4/5/3	3/4/3

Difference between groups were systematically unclear at baseline (phase I). Within-group difference in the change *vs*. Baseline

* *very likely*

** *almost certain*. Between-group difference in the change from Baseline *vs*. control

#*very likely*

*##almost certain*. f-OR: functional overreaching.

### Cardiac Measures

**Fig 3:**
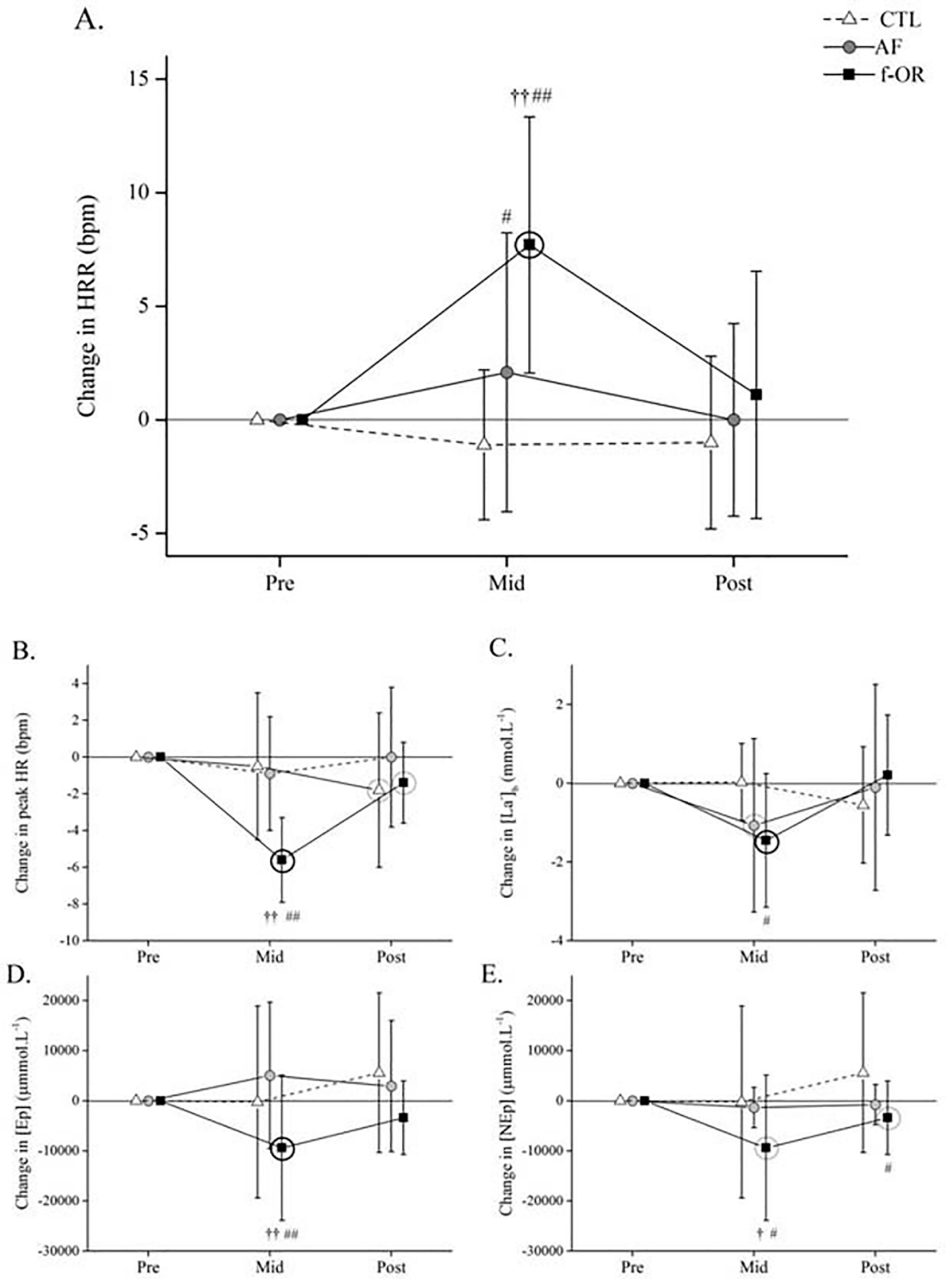
Changes in heart rate recovery (HRR, A), peak HR (B), peak blood lactate concentration ([La^-^]_,_ C), peak plasma ephinephrine concentration ([Ep], D) and peak plasma norepinephrine concentration ([NEp], E) during the maximal incremental cycling test (mean ± 90% CI). Grey and black circles around symbols denote likely (*i*.*e*., 75%–95% chances that the true value of the statistic is practically meaningful) and very likely to almost certain (*i*.*e*., > 95% chances that the true value of the statistic is practically meaningful) within-condition difference from baseline (Pre), respectively. Between-group difference in change from Pre vs. control, #likely; ## very likely to almost certain. Between-group difference in change from Pre vs. AF, †likely; †† very likely to almost certain. AF: acute fatigue; f-OR: functional overreaching.

Heart rate (HRR, HR_peak_) changes throughout the protocol in the three groups are presented in (Fig 3A and 3B, S2 Fig).

#### HR_peak_

*Overload period*: The f-OR group demonstrated an almost certain large decrease in HR_peak_ (182 ± 5 vs 176 ± 6 bpm at Pre and Mid, respectively). In contrast, the effect of the training period was unclear for both the control group (185 ± 5 vs 185 ± 5 bpm at Pre and Mid, respectively) and the AF group (182 ± 10 vs 181 ± 9 bpm at Pre and Mid, respectively). The f-OR group demonstrated an almost certain large greater decrease in HR_peak_ than the control and AF groups, respectively. In contrast, the difference in change between the control and AF groups was unclear.

*Overall protocol*: Both control (185 ± 5 vs 183 ± 10 bpm at Pre and Post, respectively) and f-OR groups (182 ± 6 vs 180 ± 5 bpm at Pre and Post, respectively) demonstrated a likely small decrease in HR_peak_. In contrast, changes were unclear in the AF group (182 ± 10 vs 182 ± 10 bpm at Pre and Post, respectively). All the differences in change between Pre and Post between the three groups were unclear.

#### HRR

*Overload period*: The f-OR group showed an almost certain very large increase in HRR (38 ± 11 vs 45 ± 11 bpm at Pre and Mid, respectively). Conversely, both control (35 ± 10 vs 34 ± 12 bpm at Pre and Mid, respectively) and AF groups showed unclear changes (40 ± 8 vs 42 ± 10 bpm at Pre and Mid, respectively). The f-OR group demonstrated an almost certain very large and a very likely large greater increase in HRR than the control and AF groups, respectively. Despite the magnitude of the effect being reduced, the between-group differences in change remained after controlling for changes in HR_peak_, with a 86% and 99% chance to observe a faster HRR in the f-OR group than in the control (very large effect) and AF (moderate effect) groups, respectively. The control group demonstrated a likely moderate difference in HRR change with the AF group.

*Overall protocol*: The changes in HRR were unclear for the control (35 ± 10 vs 34 ± 8 bpm at Pre and Post, respectively), AF (40 ± 8 vs 40 ± 9 bpm at Pre and Post, respectively) and f-OR groups (38 ± 11 vs 39 ± 8 bpm at Pre and Post, respectively). The differences in change between the three groups were systematically unclear.

### Blood parameters

Blood parameters changes ([La^-^], [Ep] and [NEp]) throughout the protocol in the three groups are depicted in (Fig 3C, 3D and 3E, S2 Fig).

#### Blood lactate concentration ([La-])

*Overload period*: The f-OR group demonstrated a very likely large decrease (12.4 ± 2.5 vs 10.9 ± 2.4 mmol·L^-1^ at Pre and Mid, respectively). Similarly, a likely moderate decrease was reported in the AF group (12.3 ± 1.9 vs 11.4 ± 2.0 mmol·L^-1^ at Pre and Mid, respectively), while the control group showed an unclear change during the same period (12.4 ± 1.4 vs 12.4 ± 0.9 mmol·L^-1^ at Pre and Mid, respectively). The f-OR group demonstrated a very likely large greater decrease than the control group but the difference in change was unclear with the AF group. The difference in change between the control and the AF groups was also unclear.

*Overall protocol*: The change in [La^-^] were unclear for the three groups. Similarly, the differences in change between the three groups were unclear.

*Overload period*: The f-OR group demonstrated a likely small decrease in peak [NEp] (42452 ± 18492 vs 33065 ± 10716 μmol·L^-1^ at Pre and Mid, respectively), while unclear changes were reported in both control (45226 ± 20996 vs 44998 ± 17657 μmol·L^-1^ at Pre and Mid) and AF groups (38449 ± 15085 vs 43509 ± 15107 μmol·L^-1^ at Pre and Mid, respectively). The f-OR group demonstrated a likely small greater decrease in peak [NEp] than both control and AF groups. The f-OR group demonstrated an almost certain moderate decrease in peak [Ep] (13402 ± 12128 vs 5476 ± 5515 μmol·L^-1^ at Pre and Mid, respectively) while the changes were unclear in the two other groups (7971 ± 4341 vs 7627 ± 4933 μmol·L^-1^ at Pre and Mid, respectively for control group; 9761 ± 7536 vs 8424 ± 5399 μmol·L^-1^ at Pre and Mid, respectively for AF group). The f-OR group demonstrated a very likely moderate greater decrease in peak [Ep] than the control and the AF groups. Conversely, the difference in change between the control and AF groups were unclear.

*Overall protocol*: The control group demonstrated a possible small increase in peak [NEp] (45226 ± 20996 vs 50824 ± 22552 μmol·L^-1^ at Pre and Post, respectively) and the f-OR group showed a likely small decrease (42452 ± 18492 vs 39058 ± 19358 μmol·L^-1^ at Pre and Post). Change in peak [NEp] was unclear in the AF group (38449 ± 15085 vs 41393 ± 14128 μmol·L^-1^ at Pre and Post, respectively). The between-group differences in change in peak [NEp] were likely small between control and f-OR groups. All the other differences in change between the three groups in peak [NEp] were unclear. The change in peak [Ep] was unclear in the three groups (13402 ± 12128 vs 12016 ± 9420 μmol·L^-1^ at Pre and Post, respectively for f-OR group; 9761 ± 7536 vs 8995 ± 6415 μmol·L^-1^ at Pre and Post, respectively for AF group; 7971 ± 4341 vs 8338 ± 4402 μmol·L^-1^ at Pre and Post, respectively for the control group). The between-group differences in change in peak [Ep] were also systematically unclear.

### Correlations between changes in HRR, performance, HR_peak_ and peak metabolites concentrations

When considering all data from the 31 participants during the overload training period together, there was a moderate negative correlation between ΔHRR, change in performance (-0.46, CI: -0.67; -0.17) and ΔHR_peak_ (-0.35, CI: -0.59; -0.05). There was also systematic small correlations between ΔHRR and Δ[La^-^] (-0.23, CI: -0.51; 0.09), Δ[Ep] (-0.25, CI: -0.53; 0.07) and Δ[NEp] (-0.27, CI: -0.54; 0.05).

## Discussion

This study provides new information demonstrating that f-OR is associated with a faster HRR in trained triathletes. Specifically, we observed that the decrease in HR during the first minute after cessation of exercise at maximal aerobic power was transiently accelerated in triathletes who became f-OR after a 3-wk overload training period; however, this change was reversed after a 2-wk taper. These findings demonstrate that a faster HRR is associated with both improved fitness as well as overreaching, and confirm that single physiological markers should not be used to monitor the responses to endurance training. Furthermore, the results also suggest that the cardiac response in the f-OR group is induced, at least partly, by a decrease in the central command related to reduced physical PPO (i.e. a lower peak HR), and by a reduced chemoreflex activity resultant from a lower post-exercise accumulation of stress metabolites (i.e. decreases in blood lactate, epinephrine and norepinephrine).

The main finding of the present study was that the f-OR athletes demonstrated a very large increase in HRR following 3-wk of overload training, whilst there were no clear changes in HRR in either the control or AF groups. We also observed a moderate negative correlation between PPO changes and HRR responses during the overload period, providing additional support for using HRR as a specific marker of f-OR in endurance athletes. Whilst differences in methodologies make accurate comparisons with previous studies difficult, the present findings are similar to several previous studies that have shown altered HR kinetics following f-OR in endurance athletes. For example, Urhausen et al. [26] showed that HR was reduced 5 minutes after completing a time-to-exhaustion at 110% of the anaerobic threshold, in f-OR endurance athletes compared to control counterparts. These authors also reported reduced peak HR values during the same period but did not directly report the changes in HRR, making it difficult to compare with the present results. Similarly, Lamberts et al. [15] observed a reproducible ~7 bpm increase in HRR in a world-class cyclo-cross athlete at the end of two distinct overload training periods associated with early signs of f-OR (*i*.*e*. very high perceived fatigue and systematic reduced HR values at exercise). Unfortunately however, no performance test was conducted, making it difficult to interpret HRR response in the context of true f-OR. Finally, both Dupuy et al. [12] and Thomson et al. [13] reported an increase in HRR in groups of endurance athletes demonstrating a decrease in mean performance after a two-week heavy training load. Nevertheless, these studies did not reported a decrease in individual performance in all participants in response to overload training, making it difficult to delineate the effects of overload training effect from true f-OR. Taken collectively, these results demonstrate that faster HRR is not always associated with better physical performance. This is supported by a recent report of a slower HRR during a taper being associated with improved marathon performance [27]. The lack of substantial change in HRR in the AF group in the present study despite improved performance also suggested that HRR response may not systematically follow the change in the performance level of endurance athletes. Therefore, we suggest that the interpretation of changes in HRR should never be performed in isolation but always be made in the context of the specific phase training (i.e. normal training, overload, taper etc.), the perceived fatigue level of athletes and the performance response.

Since HRR is an objective measure, not easily manipulated, non-invasive, inexpensive and relatively easy to assess, it meets the suggested criteria for appropriate tools for monitoring training, when interpreted in the context of the athletes’ fatigue state and the performance response. The present results show that HRR measures can be used to assess how athletes are adapting to endurance training. Indeed, in the present study we observed faster HRR in the f-OR group only, despite both the AF and f-OR groups competing the same heavy load training. Additionally, whilst the perceptions of fatigue increased for both the AF and f-OR groups following the overload period, the combination of both a faster HRR and elevated fatigue identified the f-OR athletes. Moreover, the restoration of HRR to its baseline value at the end of the taper phase, when the signs of f-OR had dissipated, shows that this measure is sensitive to changes in the athletes training state.

Previous studies have shown cardiac alterations both during exercise [5–7, 9] and during the post-exercise recovery period in f-OR endurance athletes. In agreement with these studies we also observed altered HR responses with f-OR. There are several possible explanations for the faster HRR observed in the f-OR group at the end of the overload period in this study. These are: 1) a decrease in the central command related to reduced physical performance, 2) a lower accumulation of blood borne stress metabolites (i.e. plasma catecholamines, lactate, etc.); and/or, 3) a change in the autonomic nervous control during the immediate post-exercise recovery period [11]. The present findings support the two first hypotheses and do not exclude the possibility of alterations in the nervous control upon the heart. Given the moderate negative correlations reported between ΔHRR and changes in both PPO and peak HR during the overload training period, a reduced central command may explain the acceleration in HRR in the f-OR athletes. Nevertheless, the persistence of a larger increase in HRR in the f-OR group while controlling for ΔHR_peak_ suggests that other mechanisms may be involved. In fact, the hypothesis of a reduced chemoreflex activity in the f-OR group was also supported by the decrease in peak blood lactate and plasma catecholamines concentrations in the f-OR group only. Additionally, the small negative correlations observed between ΔHRR and the changes in each blood metabolites (i.e. Δ[La^-^], Δ[Ep], Δ[NEp]) during the overload period also support this hypothesis. Finally, since the parasympathetic nervous system remains active during exercise, including at maximal intensity [28], the hypothesis of a vagal hyperactivity could also explain the altered cardiac response during the post-exercise recovery period in f-OR athletes. Indeed, both Le Meur et al. [29] and Hedelin et al. [30] have both reported a progressive increase in the parasympathetic activity of resting HR in endurance athletes with f-OR. Further investigations involving autonomic blockades are required to test whether the faster HRR associated with f-OR in endurance athletes may be also related to changes in the direct autonomic nervous action upon the heart.

### Limitations

Because the f-OR subjects were selected on the basis of their decreased performance at the end of the overload period, it is acknowledged that the subdivision of the participants employed in the present study arises a risk of confounding effect when considering the immediate post-exercise physiological response. Despite the present results persisted while controlling for ΔHR_peak_, further research investigating the HRR response and the associated metabolic response at submaximal intensities are then required to confirm the present findings.

**Fig 4:**
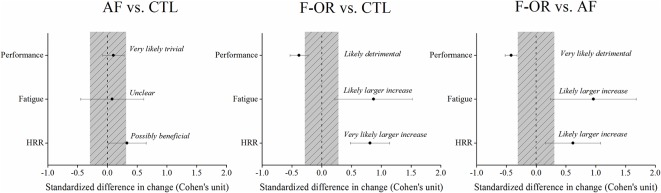
Between-group difference in changes in peak power output, perceived fatigue and HRR during the overload training period (bars indicate uncertainty in the true mean changes with 90% confidence intervals). Trivial area was calculated from the smallest worthwhile change (see methods). AF: acute fatigue; f-OR: functional overreaching.

The present results provide new information demonstrating a faster HRR after a maximal incremental cycling test in trained triathletes who developed f-OR during an overload training program, and suggest that when interpreted in the context of the athletes’ fatigue state and training phase HRR may be a practical tool for monitoring training. From a practical perspective, these findings highlight the importance of monitoring both HRR and mood states together to accurately identify endurance athletes suspected of f-OR during heavy training periods (Fig 4). Without excluding the hypothesis of potential changes in autonomic control, the present results suggest that the faster HRR associated with f-OR is induced, at least partly, by a decrease in the central command related to reduced physical performance and by a reduced chemoreflex activity due to lower post-exercise accumulation of stress system metabolites. Overall, these findings suggests that faster HRR does not systematically predict better physical performance and that its interpretation should always be made in relation to the specific training phase of an endurance training program, the perceived fatigue level of athletes and the performance response. Further research is required to determine whether these results can be reproduced at submaximal intensities, in different sports and in female athletes.

## Supporting Information

S1 Fig Performance and perceived fatigue data. (XLSX)

S2 Fig Heart rate and blood parameters data. (XLSX)

## References

[1] Meeusen R, Duclos M, Foster C, Fry A, Gleeson M, Nieman D, et al Prevention, diagnosis, and treatment of the overtraining syndrome: joint consensus statement of the European College of Sport Science and the American College of Sports Medicine. Medicine and science in sports and exercise. 2013;45(1):186–205. Epub 2012/12/19. 10.1249/MSS.0b013e318279a10a . 23247672

[2] Aubry A, Hausswirth C, Louis J, Coutts AJ, Le Meur Y. Functional overreaching: the key to peak performance during the taper? Medicine and science in sports and exercise. 2014;46(9):1769–77. Epub 2014/08/19. 10.1249/MSS.0000000000000301 . 25134000

[3] Halson SL, Jeukendrup AE. Does overtraining exist? An analysis of overreaching and overtraining research. Sports medicine. 2004;34(14):967–81. Epub 2004/12/02. . 15571428 10.2165/00007256-200434140-00003

[4] Buchheit M. Monitoring training status with HR measures: do all roads lead to Rome? Frontiers in physiology. 2014;5:73 Epub 2014/03/01. 10.3389/fphys.2014.00073 24578692 PMC3936188

[5] Le Meur Y, Hausswirth C, Natta F, Couturier A, Bignet F, Vidal PP. A multidisciplinary approach to overreaching detection in endurance trained athletes. Journal of applied physiology. 2013;114(3):411–20. Epub 2012/12/01. 10.1152/japplphysiol.01254.2012 . 23195630

[6] Bosquet L, Leger L, Legros P. Blood lactate response to overtraining in male endurance athletes. European journal of applied physiology. 2001;84(1–2):107–14. Epub 2001/06/08. 10.1007/s004210000343 . 11394238

[7] Hedelin R, Kentta G, Wiklund U, Bjerle P, Henriksson-Larsen K. Short-term overtraining: effects on performance, circulatory responses, and heart rate variability. Medicine and science in sports and exercise. 2000;32(8):1480–4. Epub 2000/08/19. . 10949015 10.1097/00005768-200008000-00017

[8] Lehmann M, Dickhuth HH, Gendrisch G, Lazar W, Thum M, Kaminski R, et al Training-overtraining. A prospective, experimental study with experienced middle- and long-distance runners. International journal of sports medicine. 1991;12(5):444–52. Epub 1991/10/01. 10.1055/s-2007-1024711 . 1752709

[9] Le Meur Y, Louis J, Aubry A, Gueneron J, Pichon A, Schaal K, et al Maximal exercise limitation in functionally overreached triathletes: role of cardiac adrenergic stimulation. Journal of applied physiology. 2014;117(3):214–22. Epub 2014/06/14. 10.1152/japplphysiol.00191.2014 . 24925979

[10] Daanen HA, Lamberts RP, Kallen VL, Jin A, Van Meeteren NL. A systematic review on heart-rate recovery to monitor changes in training status in athletes. International journal of sports physiology and performance. 2012;7(3):251–60. Epub 2012/02/24. . 22357753 10.1123/ijspp.7.3.251

[11] Buchheit M, Papelier Y, Laursen PB, Ahmaidi S. Noninvasive assessment of cardiac parasympathetic function: postexercise heart rate recovery or heart rate variability? American journal of physiology Heart and circulatory physiology. 2007;293(1):H8–10. Epub 2007/03/27. 10.1152/ajpheart.00335.2007 . 17384128

[12] Dupuy O, Bherer L, Audiffren M, Bosquet L. Night and postexercise cardiac autonomic control in functional overreaching. Applied physiology, nutrition, and metabolism = Physiologie appliquee, nutrition et metabolisme. 2013;38(2):200–8. Epub 2013/02/27. 10.1139/apnm-2012-0203 . 23438233

[13] Thomson R, Bellenger C, Howe P, Karavirta L, Buckley J. Improved heart rate recovery despite reduced exercise performance following heavy training: A within-subject analysis. Journal of science and medicine in sport / Sports Medicine Australia. 10.1016/j.jsams.2015.02.010 25797181

[14] Borresen J, Lambert MI. Changes in heart rate recovery in response to acute changes in training load. European journal of applied physiology. 2007;101(4):503–11. Epub 2007/08/10. 10.1007/s00421-007-0516-6 . 17687564

[15] Lamberts RP, Rietjens GJ, Tijdink HH, Noakes TD, Lambert MI. Measuring submaximal performance parameters to monitor fatigue and predict cycling performance: a case study of a world-class cyclo-cross cyclist. European journal of applied physiology. 2010;108(1):183–90. Epub 2009/11/19. 10.1007/s00421-009-1291-3 . 19921241

[16] Bosquet L, Montpetit J, Arvisais D, Mujika I. Effects of tapering on performance: a meta-analysis. Medicine and science in sports and exercise. 2007;39(8):1358–65. Epub 2007/09/01. 10.1249/mss.0b013e31806010e0 . 17762369

[17] Mac Nair D, Lorr M, Droppleman L. Profile of Mood States Manual. San Diego: Educational and Industrial Testing Service; 1971 p. 27.

[18] Dupuy O, Lussier M, Fraser S, Bherer L, Audiffren M, Bosquet L. Effect of overreaching on cognitive performance and related cardiac autonomic control. Scandinavian journal of medicine & science in sports. 2014;24(1):234–42. Epub 2012/04/28. 10.1111/j.1600-0838.2012.01465.x . 22537000

[19] Howley ET, Bassett DR Jr, Welch HG. Criteria for maximal oxygen uptake: review and commentary. Medicine and science in sports and exercise. 1995;27(9):1292–301. Epub 1995/09/01. . 8531628

[20] Pyne DB, Boston T, Martin DT, Logan A. Evaluation of the Lactate Pro blood lactate analyser. European journal of applied physiology. 2000;82(1–2):112–6. Epub 2000/07/06. 10.1007/s004210050659 . 10879451

[21] Charloux A, Lonsdorfer-Wolf E, Richard R, Lampert E, Oswald-Mammosser M, Mettauer B, et al A new impedance cardiograph device for the non-invasive evaluation of cardiac output at rest and during exercise: comparison with the "direct" Fick method. European journal of applied physiology. 2000;82(4):313–20. Epub 2000/08/25. 10.1007/s004210000226 . 10958374

[22] Dupuy O, Mekary S, Berryman N, Bherer L, Audiffren M, Bosquet L. Reliability of heart rate measures used to assess post-exercise parasympathetic reactivation. Clinical physiology and functional imaging. 2012;32(4):296–304. Epub 2012/06/12. 10.1111/j.1475-097X.2012.01125.x . 22681607

[23] Borg G. Perceived exertion as an indicator of somatic stress. Scandinavian journal of rehabilitation medicine. 1970;2(2):92–8. Epub 1970/01/01. . 5523831

[24] Hopkins WG, Marshall SW, Batterham AM, Hanin J. Progressive statistics for studies in sports medicine and exercise science. Medicine and science in sports and exercise. 2009;41(1):3–13. Epub 2008/12/19. 10.1249/MSS.0b013e31818cb278 . 19092709

[25] Batterham AM, Hopkins WG. Making meaningful inferences about magnitudes. International journal of sports physiology and performance. 2006;1(1):50–7. Epub 2006/03/01. . 19114737

[26] Urhausen A, Gabriel HH, Weiler B, Kindermann W. Ergometric and psychological findings during overtraining: a long-term follow-up study in endurance athletes. International journal of sports medicine. 1998;19(2):114–20. Epub 1998/04/30. 10.1055/s-2007-971892 . 9562220

[27] Hug B, Heyer L, Naef N, Buchheit M, Wehrlin JP, Millet GP. Tapering for marathon and cardiac autonomic function. International journal of sports medicine. 2014;35(8):676–83. Epub 2014/03/07. 10.1055/s-0033-1361184 . 24595813

[28] White DW, Raven PB. Autonomic neural control of heart rate during dynamic exercise: revisited. The Journal of physiology. 2014;592(Pt 12):2491–500. Epub 2014/04/24. 10.1113/jphysiol.2014.271858 24756637 PMC4080933

[29] Le Meur Y, Pichon A, Schaal K, Schmitt L, Louis J, Gueneron J, et al Evidence of parasympathetic hyperactivity in functionally overreached athletes. Medicine and science in sports and exercise. 2013;45(11):2061–71. Epub 2013/10/19. 10.1249/MSS.0b013e3182980125 . 24136138

[30] Hedelin R, Wiklund U, Bjerle P, Henriksson-Larsen K. Cardiac autonomic imbalance in an overtrained athlete. Medicine and science in sports and exercise. 2000;32(9):1531–3. Epub 2000/09/20. . 10994900 10.1097/00005768-200009000-00001

